# Determining the fibrillar orientation of bast fibres with polarized light microscopy: the modified Herzog test (red plate test) explained

**DOI:** 10.1111/jmi.12079

**Published:** 2013-09-10

**Authors:** E HAUGAN, B HOLST

**Affiliations:** Department of Physics and Technology, University of BergenBergen, Norway

**Keywords:** Fibrillar orientation, Herzog test, plant fibre identification, polarized light microscopy, red plate test

## Abstract

The identification of bast fibre samples, in particular, bast fibres used in textiles, is an important issue in archaeology, criminology and other scientific fields. One of the characteristic features of bast fibres is their fibrillar orientation, referred to as Z- or S twist (or alternatively right- and left-handed fibres). An empirical test for determining the fibrillar orientation using polarized light microscopy has been known in the community for many years. It is referred to as the modified Herzog test or red plate test. The test has the reputation for never producing false results, but also for occasionally not working. However, so far, no proper justification has been provided in the literature that the ‘no false results’ assumption is really correct and it has also not been clear up till now, why the method sometimes does not work. In this paper, we present an analytical model for the modified Herzog test, which explains why the test never gives a false result. We also provide an explanation for why the Herzog test sometimes does not work: According to our model, the Herzog test will not work if none of the three distinct layers in the secondary cell wall is significantly thicker than the others.

## Introduction

**Fig 1:**
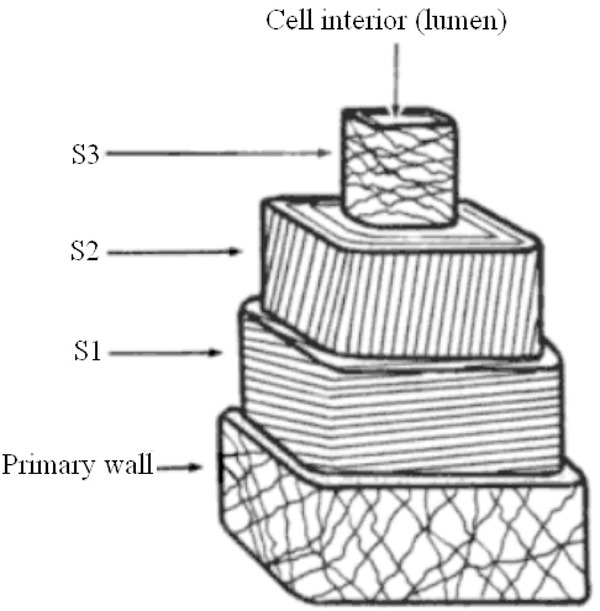
Diagram of a typical textile bast fibre cell showing the fibrillar orientations of the sublayers. S2 is here shown with Z-twist. Edited from Burgess (1985).

The identification of fibres, in particular, textile fibres, is important in several scientific fields (Goodway, 1987; Petraco & Kubik, 2004; Brettell *et al.*, 2011). Although it is relatively simple to separate between plant and animal fibres (animal fibres have scales), it is much more difficult to identify different types of plant fibres correctly (Catling & Grayson, 2007; Bergfjord *et al.*, 2010). Most plant fibres used for textile production (apart from cotton) are bast fibres. The term bast is commonly used to describe bundles of tightly joint fibre cells found in the stem of plants like hemp, flax, jute, ramie and nettle or in the inner bark of wood. Each bast fibre cell consists of a cell wall, which surrounds an empty space (lumen). The cell wall is separated into two parts: the primary (outermost) and the secondary wall. The cell wall contains so-called macrofibrils, which in turn are made up of microfibrils. The microfibrils consist of chains of cellulose that are birefringent. In the primary wall, the microfibrils are arranged randomly, but with a generally longitudinal orientation in the outer part. In the secondary wall, the microfibrils are arranged in a corkscrew (helical) fashion, winding around the longitudinal axis of the fibre (Beck, 2005). In many plants the secondary wall consists of three distinct layers, commonly known as S1, S2 and S3, as shown in Figure 1. The microfibrils in these three sublayers can twist in different directions. It is the helical orientation of the microfibrils found in the thickest region of the secondary wall, which is used to designate the overall fibrillar orientation of a fibre as either Z or S-twist (right- or left-handed fibre). The spiral angle of the dominating layer is known as the *fibrillar angle* (ϕ) or twist angle of the fibre. Fibrillar orientation is a characteristic feature for a species and serves as an aid for identification (Herzog, 1955). For example, knowledge about the fibrillar orientation of a fibre and the presence of calcium oxalate crystals in the associated tissue makes it possible to conclusively distinguish nettle/ramie fibres from hemp, flax and jute (Bergfjord & Holst, 2010; Bergfjord *et al.*, 2012). The composition of fibre cells in most common bast textile plants are in fact very similar. For example, in flax, hemp and ramie S1 is Z-twist and S2 is S-twist. S3 is Z-twist in flax, while in ramie and hemp the microfibrils in S3 are almost parallel to the fibre axis (Harris, 1954; Meredith, 1956; Lewin, 2006). However, the relative thickness of S1, S2 and S3 is different, making hemp overall Z-twist and flax and ramie S-twist. In flax, ramie and hemp, the magnitudes of the fibrillar angles are 6.5°, 7.0°and 7.5°, respectively. Jute is Z-twist (Chakravarty & Hearle, 1967; Lewin, 2006) and nettle is S-twist (Bergfjord & Holst, 2010).

The most widespread method for determining the fibrillar orientation of a textile bast fibre is the so-called twist test or drying twist test (Herzog, 1985; Goodway, 1987): A wet fibre, fixed at one end, will coil into a corkscrew (twist) when drying. The direction of the twist will normally be equivalent to the fibrillar orientation. The drying twist test can only be applied when longer fibre samples are available. In some cases, for example, if the fibre is damaged, it may not work or even give a wrong result.

Luniak (1953), Goodway (1987), Valaskovic (1991), Batchelor *et al*. (1997), Petraco & Kubik (2004) and others describe a method, based on original work by Herzog (1955), using polarized light microscopy to determine the fibrillar orientation of bast fibres. This technique has come to be known as the modified Herzog test or red-plate test. In the modified Herzog test, white light from the polarizer enters the birefringent sample. With the sample oriented at extinction (see next section) a 530-nm full wave compensator (also referred to as a retardation plate or red plate) is inserted at 45° to the crossed polars. It is claimed that this will produce additive or subtractive compensation

which causes the fibre to turn either slightly blue or yellow. The colour change is said to depend on whether the fibre is S- or Z-twist. A Z-twist fibre is said to turn yellow when parallel to the polarizer and blue when parallel to the analyser, while for a S-twist fibre the situation is exactly opposite. The modified Herzog test can also be used to distinguish between bast fibres and other plant fibres. For example, in cotton, a seed fibre, the microfibrils change their twist directions at short intervals (Peterlin & Ingram, 1970; Goodway, 1987) so that it will normally not be possible to observe any extinction and when using the compensator plate a rapid colour change along the the fibre will be observed (see Fig. 3). Of the authors listed above only Valaskovic (Valaskovic, 1991) provides any formal treatment of the Herzog test. Similar to us, he suggests that it can be modelled using the Jones Matrix formalism with each cell wall being treated as a linear retarder. He provides computer simulations for the light intensity in various configurations to illustrate this, but he does not derive an analytical expression for the light intensity distribution as we do. Further practical experience shows that the Herzog test does not always work and none of the authors listed above provide any explanation as to when the method works and when not.

**Fig 2:**
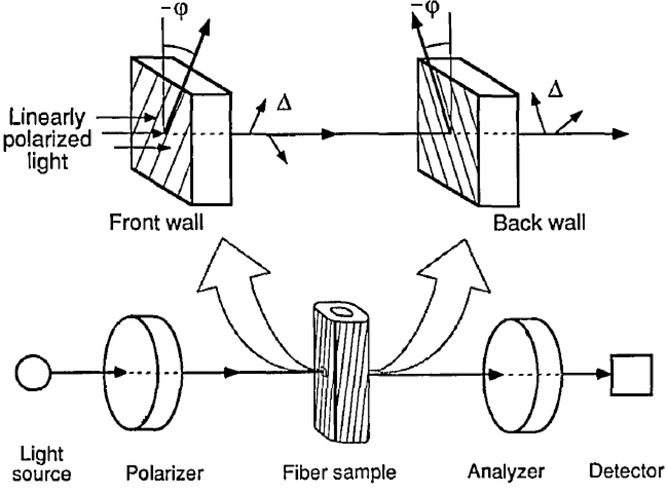
The front and back of the cell wall are described as linear retarders with the same relative retardation and opposite angles of orientation. Adapted from Ye *et al*. (1994). This set-up corresponds to the modified Herzog test set-up except that here an additional 530 nm full wave compensator is inserted between the polarizer and analyser.

Ye *et al*. (1994) have developed a method, shown in Figure 2, using polarized light microscopy for determining the exact fibrillar orientation of various kinds of wood fibres. The set-up is similar to the set-up used in the modified Herzog test, apart from the additional full wave compensator used in the modified Herzog test. Ye and coworkers have also developed an analytical model to describe their results. In this paper, we have adapted their model for use with textile plant bast fibres and present a first analytical model for the modified Herzog test. The basic assumption of our model is that for textile plant bast fibres, one of the secondary wall layers (S1, S2 or S3) is so much thicker that the others can be ignored. This is normally the case (Chakravarty & Hearle, 1967; Lewin, 2006).

## Analytical model for the modified Herzog test

When polarized light passes through a uniaxially birefringent medium it gets split into ordinary (*O*) and extraordinary (*E*) rays whose electric field vectors oscillate in perpendicular planes. The *O* and *E* rays experience different refractive indices, which cause them to travel at different speeds through the medium. The difference in speed between the *E* and *O* rays gives rise to a relative retardation (RRT) given as
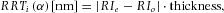
1where 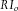 and 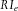 are the refractive indices of the ordinary and extraordinary rays, respectively (Murphy, 2001).

**Fig 3:**
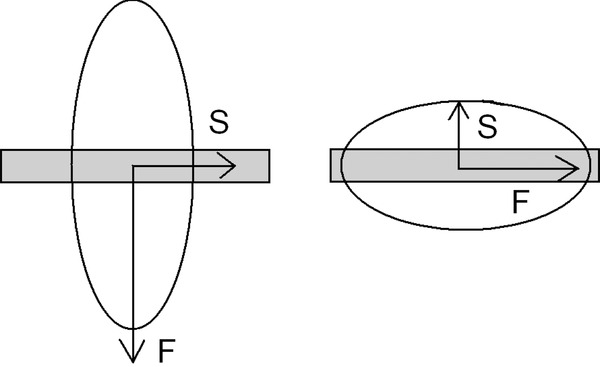
Positive (left) and negative (right) sign of elongation. S and F refer to the slow and fast rays, respectively.

For the purpose of the following discussion, consider an elongated sample of a uniaxially birefringent material. A property called the *sign of elongation* (SE) is useful in this context. If the slow ray (the one with the higher refractive index) is oriented along the geometric length of the sample, it is said to have a positive SE. This is illustrated in Figure 3, where the wavefront ellipse is shown.

**Fig 4:**
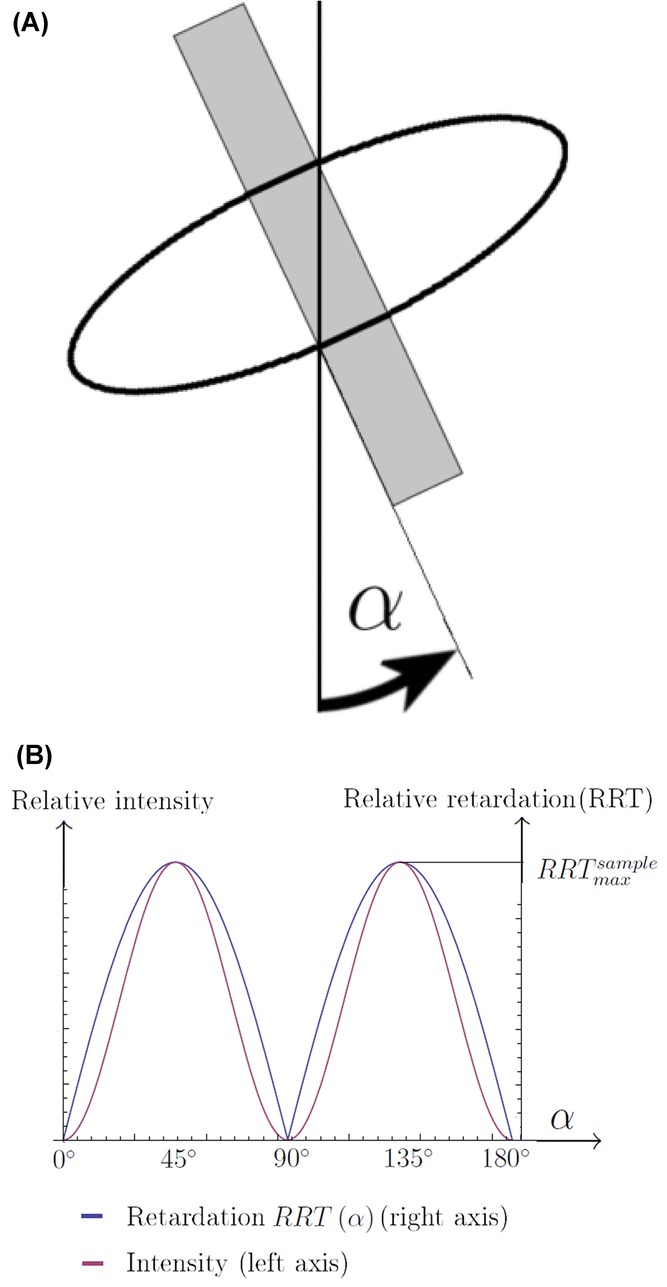
(A) The sample orientation angle α defined relative to the crossed polars. The vertical line is the analyser axis. (B) Schematic illustration of how RRT and relative intensity depend on the sample orientation angle α.

The refractive index of the E-ray, and hence the *RRT*_sample_, depends sinusoidally on the sample orientation angle relative to the crossed polars (Mea, 2005). It attains its maximum value (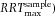 at 90° intervals. It can be shown that the intensity of light emerging from a birefringent sample also depends on the orientation of the sample. Using the notation for the sample orientation angle α, defined in Figure 4(A), maximum intensity and retardation occur at the same angles, namely when 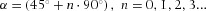, as seen in Figure 4(B) (Petraco & Kubik, 2004; Olympus Microscopy, 2010).

A special situation arises when the sample is oriented such that its optical axis is either perpendicular or parallel to the transmission axis of the polarizer. The sample is then said to be *at extinction*, where the intensity is minimum. Extinction thus occurs for 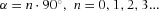


In the Herzog test, a 530-nm full wave compensator (retardation plate) is inserted into the microscope column at a fixed 45° angle to the crossed polars. A compensator consists of a birefringent material with a known RRT, here called 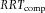. The slow axis is directed along the Northeast–Southwest direction (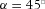 and 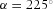).

With the compensator installed, the light passes through two birefringent media placed after one another: the compensator and the sample. In this situation, the total retardation is given as
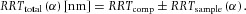
2

The sign in Eq. 2 is positive if the angle between the slow axes of the specimen and compensator is less than 90°, and negative if it is greater than 90° (Petraco & Kubik, 2004; Olympus Microscopy, 2010). Recall that, as was shown in Figure 4(B), the RRT of the specimen depends on its orientation, with maxima for 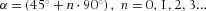


The RRT occurs for all colours of the white light. It can also be expressed as a phase shift △ for each wavelength, as given by Eq. 3 (Ye *et al.*, 1994):
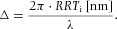
3

**Fig 5:**
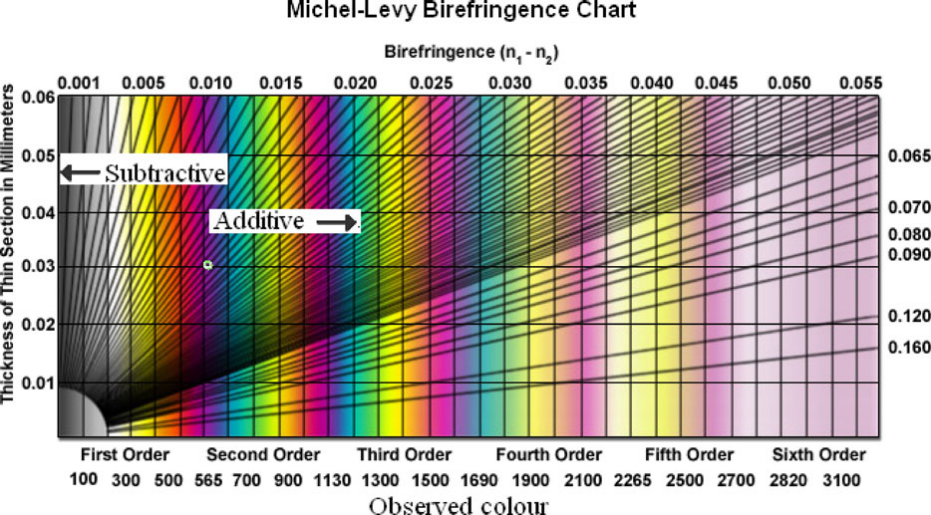
Michel–Levy birefringence chart. Edited from Olympus Microscopy (2010).

The varying phase shift for different wavelengths leads to possible destructive interference and the observation of colour, commonly visualized by a Michel–Levy birefringence chart, see (Fig. 5).

If destructive interference occurs at a wavelength shorter than that of the RRT compensator (i.e. less than 530 nm), the sign is negative and we have *subtractive compensation*. The colour which is observed through the oculars is to the left (lower order) of first-order magenta in the Michel–Levy birefringence chart. If the blocked light is of a longer wavelength than that of the compensator (i.e. longer than 530 nm), the sign in Eq. 2 is positive and *additive compensation* occurs. The colour which is observed is to the right (higher order) in the Michel–Levy plot (Murphy, 2001; Petraco & Kubik, 2004; Olympus Microscopy, 2010).

**Fig 6:**
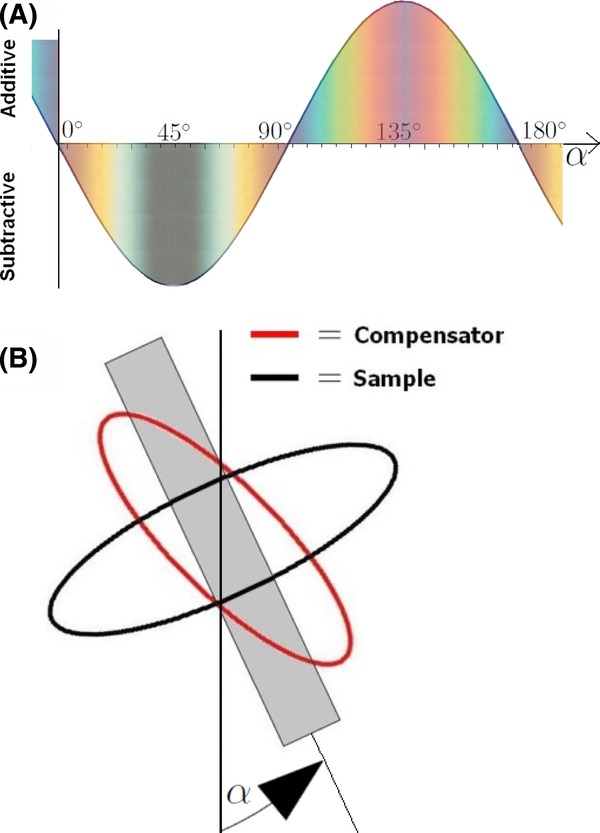
(A) The total retardation as a function of the sample orientation angle α for a sample with positive elongation. The expected observable colours are also shown, from the Michel–Levy birefringence chart (Fig. 5). Note the blue and yellow hues for α close to 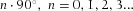. (B) Wavefront ellipses of the compensator and sample, vertical line represents the analyser axis.

In the case where the slow axes of the specimen and compensator are not exactly parallel or perpendicular (i.e. for all other angles than 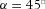 and 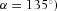, *partially* subtractive or additive compensation occurs. This effect is shown in Figure 6 for the case of a specimen with a positive SE. Observe that when the sample is rotated a few degrees away from 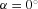 and 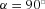, a blue or yellow shift is observed.

**Fig 7:**
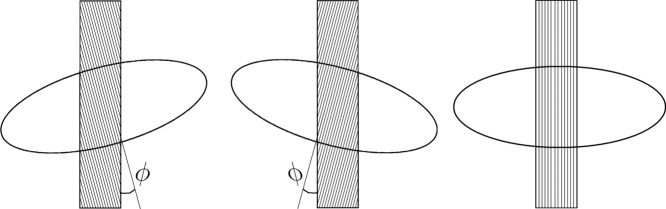
Wavefront ellipse of outer (top) layer of fibre. Left: 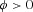 (S-twist), middle: 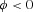 (Z-twist), right: 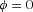 (no twist).

The model proposed by Ye *et al*. (1994) treats each cell wall (see Fig. 2), as a linear retarder. Assuming a positive SE, the wavefront ellipse associated with the top layer of the fibre (closest to the polarizer) is illustrated in Figure 7.

Jones matrix formalism can be used to make predictions about the intensity of light passing through the fibre. If each wall has a phase shift of △ and the fibrillar angle is ϕ, the whole fibre can be treated as a Solc filter of the first rank (Yariv & Yeh, 1984; Ye *et al.*, 1994). Equation 3 is written as
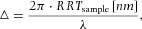
where
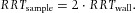


From Ye *et al*. (1994) (building on Yariv & Yeh, 1984), the transmission function 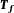 of the whole fibre can be written generally as:
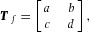
4

where
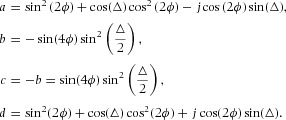


If an optical element (such as a linear retarder) is rotated by an angle α, the transmission function of the rotated element is given by
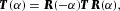
5where
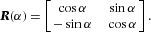
6

Therefore, the Jones matrix of the fibre, as a function of its rotation angle α with respect to the analyser (Fig. 6B), is written generally as:
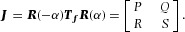


The light entering the fibre from the polarizer has Jones vector 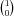. After passing through the fibre, the Jones vector of the light is 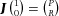. This light then encounters the analyser, whose transmission function is (Pedrotti *et al.*, 1993; Collett, 2005):
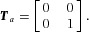
7

The Jones vector of light exiting the analyser is, therefore, 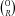. Hence, 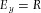 and the intensity 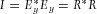. After some calculation, light passing through the analyser is found to have an intensity which depends on the fibre orientation α as follows:
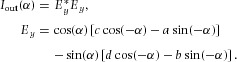
8

**Fig 8:**
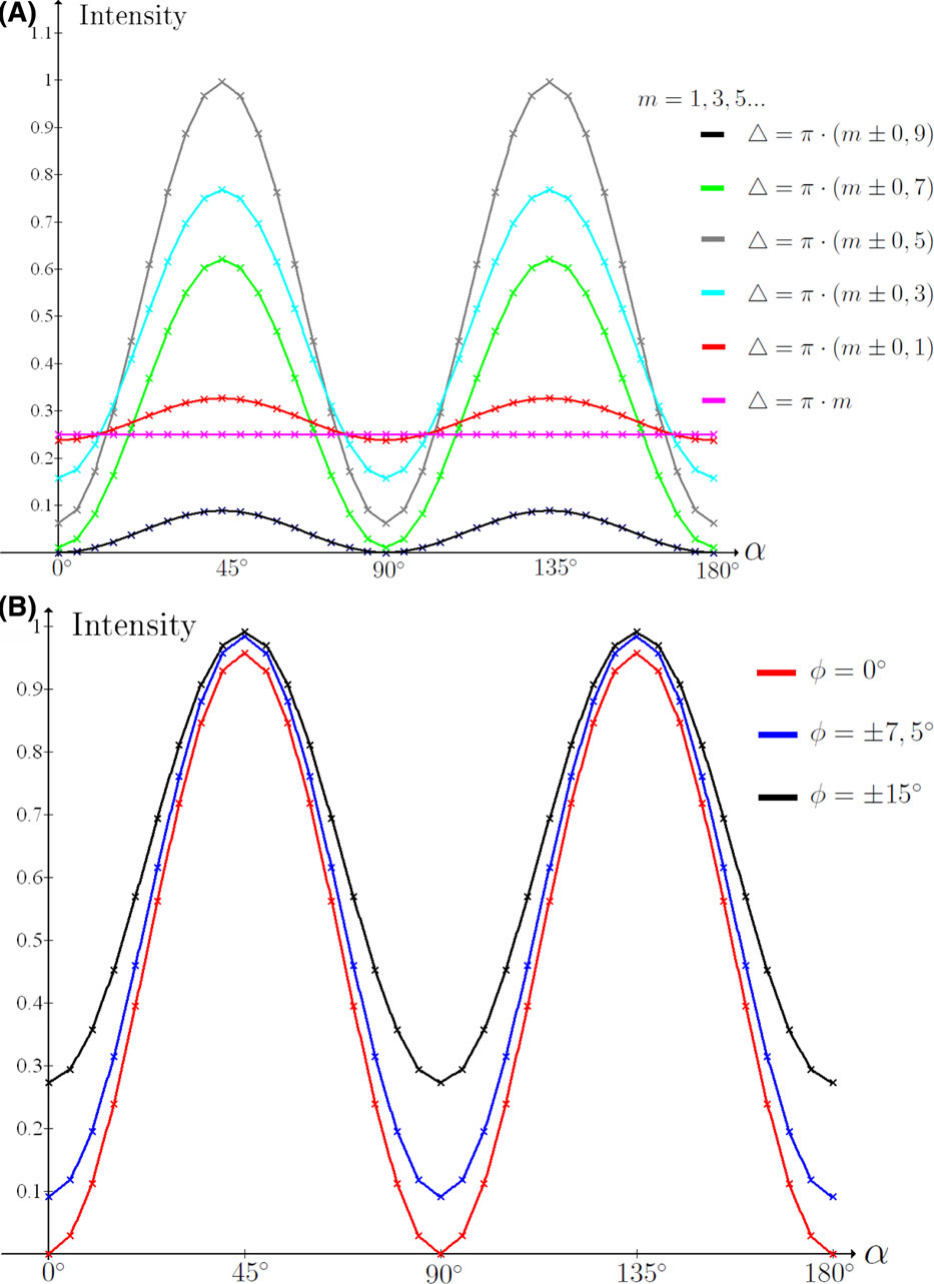
(A)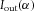 for various values of △ of the fibre. ϕ is fixed at 7.5°. (B) 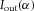 for various values of fibrillar orientation ϕ. △ of fibre is fixed. Note that, the intensity minimum for all cases occurs at 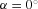
and 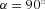.

A plot of 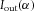 is shown in Figure 8, demonstrating how 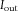 varies with ϕ and △ as well as α. As is clearly seen, 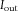 attains its minimum for 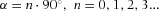 Thus, the main result from these calculations is that for all retardations and fibrillar angles of a fibre, minimum intensity is expected to occur at 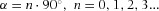.

**Fig 9:**
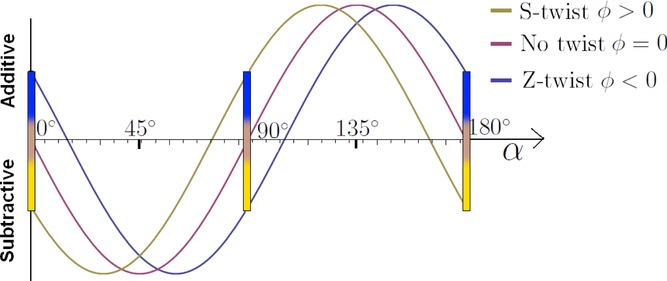
Total retardation (full wave compensator plate included) as a function of the sample orientation angle α. The coloured bars indicate expected colours. The fibre with no twist shows no blue or yellow shift when 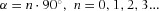 (A) 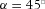. (B) 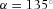.

The Herzog test uses a full wave compensator plate, which displays the retardation as a colour change as explained above. It is the manner in which the wavefront ellipse of the top wall of the fibre overlaps with that of the compensator, which determines whether additive or subtractive compensation occurs. In bast fibres, the microfibrils are oriented at a slight angle to the longitudinal axis of the fibre. Following our model this means that the wavefront ellipse of the front and back walls of the fibres will be slightly tilted with respect to the longitudinal axis, as illustrated in Figure 7. The expected colour change can be found by using the Michel–Levy birefringence chart. Figure 6 corresponds to a fibre with no twist. For Z- and S-twist fibres, the expected colours can be determined by shifting the graph in Figure 6 slightly to the right or left as illustrated in Figure 9. It can be seen that S-twist fibres are predicted to show a yellow hue for 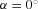 and blue for 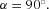 Z-twist fibres show the opposite behaviour. This is in exact agreement with the modified Herzog test.

## Experimental results

An Olympus BX-51P compound microscope equipped with a full wave compensator of wavelength 530 nm, 10× oculars and objectives of the type UIS2 Ach N, was used for all measurements presented here. Fibre samples of ramie, nettle, hemp, jute and flax were prepared according to the method described in Bergfjord & Holst (2010). It is repeated here for completeness: A fibre bundle was cut into short pieces, which were placed on a glass slide and 2–3 drops of distilled water were added in order to make it easier to separate the individual fibres. Separation was done using tweezers and with the aid of a stereo microscope. The refractive index of the mounting medium used was 1.5. A cover glass slip of a thickness matching the objectives was carefully mounted and a pencil eraser was gently pressed against the cover glass to remove any air bubbles as described by Petraco & Kubik (2004). After curing overnight the samples were ready for investigation.

### Sign of elongation (SE)

**Fig 10:**
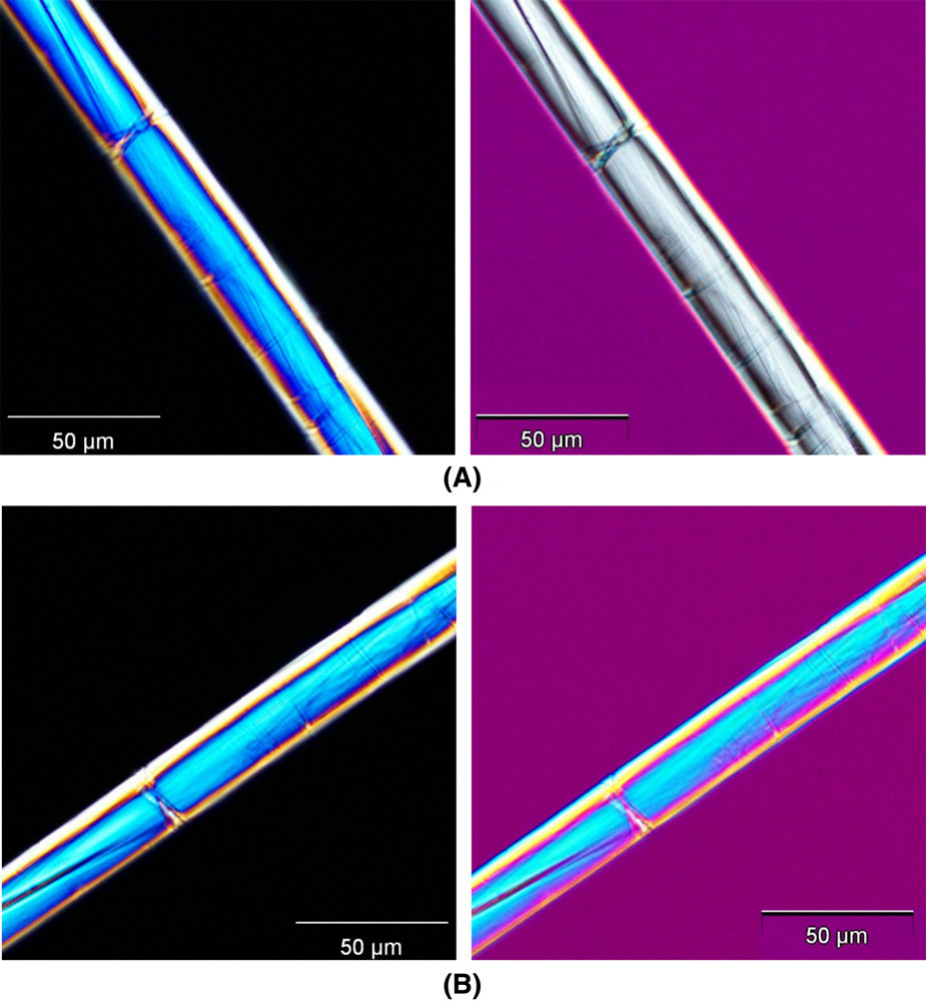
Demonstration of sign of elongation. (A) α = 45°, (B) α = 135°. Fibre is nettle. At 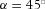 zero-order grey is clearly observed, which proves the expected positive sign of elongation (see Fig. 6A).

As a first step, the SE of the various fibre species was determined. Individual fibres of the five fibre types were examined at 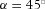 and 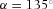. Recall from Figure 6 that if a fibre shows subtractive compensation at 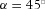
and additive compensation for 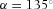, it must have a positive SE. When the compensator was inserted into the microscope column at a fixed 45° angle to the crossed polars, all fibre types gave clearly subtractive compensation at 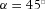 and clearly additive at 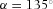 degrees. It was concluded that all the samples had a positive SE as expected. The results for a nettle sample is shown in Figure 10.

### Intensity curves

Figure 8 shows the intensity curve as a function of orientation angle for a cross-polar configuration predicted by our model. We tested the intensity curves for all fibre types. All fibres attained their minimum intensity for 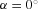 and 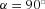, as predicted. The result for a flax sample is shown in Figure 1.

**Fig 11:**
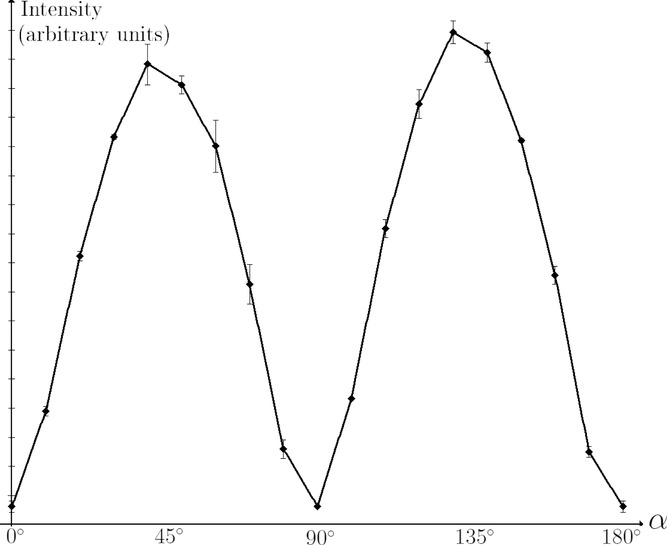
Measured intensity graph for a flax fibre (S-twist) as a function of sample orientation angle α. Note the excellent agreement with the theoretical predictions in Figure 8.

### Modified Herzog test

Fibres were investigated using the method as described by Luniak (1953) and Goodway (1987). It is repeated here for completeness: First, a single fibre was identified and brought into focus in the polarized light microscope using a 40× objective. The analyser was inserted into the microscope column in a cross-polar configuration and a fibre segment oriented to extinction (a small segment of the sample looks black). Then the compensator was inserted and the fibre was observed through the oculars to look for a colour change to either blue or yellow. When looking for colour changes, it is vital to consider only the small fibre segment which was at extinction; other parts must be ignored completely. The sample stage was then rotated by 90° and the fibre segment was observed again to look for a colour change, which should now be either yellow or blue. Results for the modified Herzog test performed on S- and Z-twist fibres (nettle and jute) is shown in Figure 2. The expected blue and yellow shifts are clearly seen.

**Fig 12:**
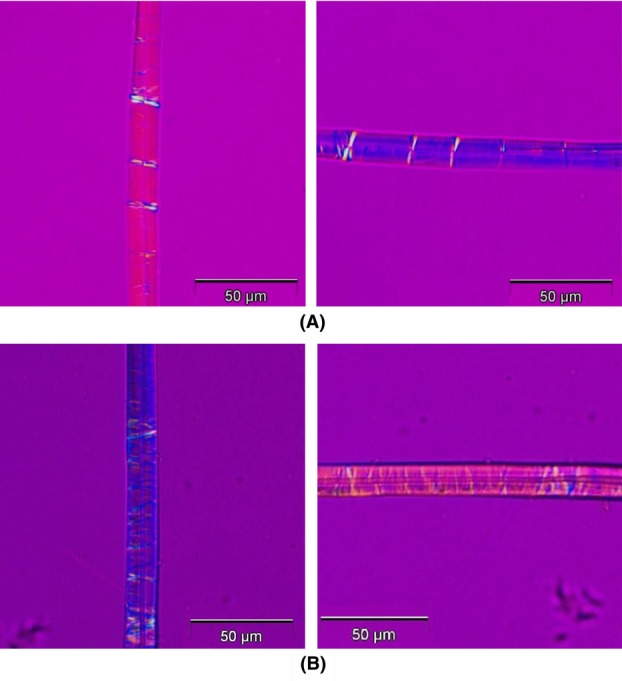
The modified Herzog test performed on (A) nettle (S-twist) and (B) jute (Z-twist). To the left the sample orientation angle 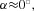 to the right the sample orientation angle 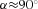. Note the excellent agreement with the theoretical predictions in Figure 9.

Several experiments were performed with each fibre type. No fibres showed the opposite colour change to what was expected. However, some fibres were observed to go less completely to extinction than others and this reduced the visibility of the colour change when the compensator was inserted. This is also in agreement with the predictions illustrated in Figure 8(A), which shows that the intensity at the minima depends on the retardation (and hence the thickness) of each wall. This can vary from fibre to fibre and even within a fibre. This is an explanation for why the modified Herzog test sometimes does not work.

### Cotton

As mentioned in the introduction, the modified Herzog test can be used to distinguish bast fibres from other textile fibres, such as the seed-fibre cotton. Cotton does not have a well-defined fibrillar angle and hence is not affected by the Herzog test, as shown in Figure 3. This image is obtained using a 20× objective. In some cases, the twist may change so rapidly that it appears as if the cotton fibre does not change at all when rotated.

**Fig 13:**
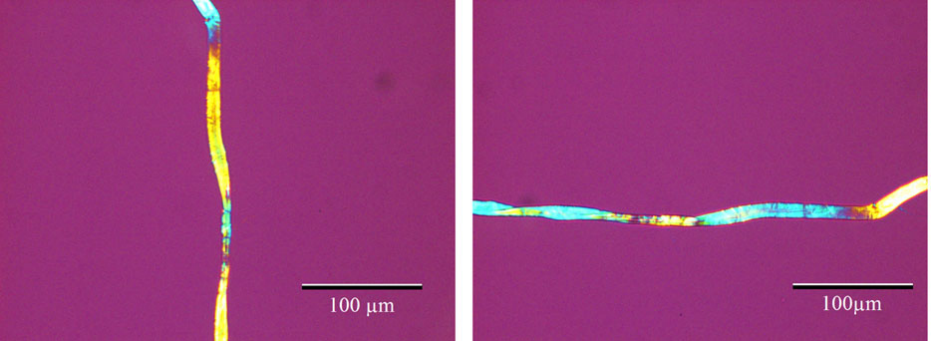
The modified Herzog test performed on cotton. Cotton is not a bast fibre with well-defined fibrillar orientation hence it will normally not be possible to bring it to extinction and it will show a rapid colour change along the fibre, regardless of the orientation angle. In some cases it may appear as if there is no colour change at all with orientation angle.

## Conclusion

We present the first analytical model for the modified Herzog test. The basic assumption of our model is that an individual fibre cell can be treated as a Solc filter of the first rank (Ye *et al.*, 1994) (Fig. 2). In addition to the theoretical work, we did a range of experiments on hemp, flax, ramie, jute and nettle fibres, some of which are shown here. Both the minimum brightness behaviour as well as the modified Herzog test predictions were confirmed by our experiments. Not all fibres showed a clear colour change. According to our model, this can be explained by varying wall thicknesses and/or fibrillar angles of the S1/S2/S3 sublayers of the secondary wall. We conclude, in agreement with established experience, that the modified Herzog test does not yield a result for all fibres, but that when it does, it is a trustworthy method for determining the fibrillar orientation.
